# Evaluation of Different Machine Learning Approaches to Predict Antigenic Distance Among Newcastle Disease Virus (NDV) Strains

**DOI:** 10.3390/v17040567

**Published:** 2025-04-14

**Authors:** Giovanni Franzo, Alice Fusaro, Chantal J. Snoeck, Aleksandar Dodovski, Steven Van Borm, Mieke Steensels, Vasiliki Christodoulou, Iuliana Onita, Raluca Burlacu, Azucena Sánchez Sánchez, Ilya A. Chvala, Mia Kim Torchetti, Ismaila Shittu, Mayowa Olabode, Ambra Pastori, Alessia Schivo, Angela Salomoni, Silvia Maniero, Ilaria Zambon, Francesco Bonfante, Isabella Monne, Mattia Cecchinato, Alessio Bortolami

**Affiliations:** 1 Department of Animal Medicine, Production and Health (MAPS), Padua University, 35020 Legnaro, Italy; mattia.cecchinato@unipd.it; 2 Division of Comparative Biomedical Sciences (DSBIO), Istituto Zooprofilattico Sperimentale delle Venezie, Viale dell’Università 10, 35020 Legnaro, Italy; afusaro@izsvenezie.it (A.F.); ambrj@hotmail.it (A.P.); aschivo@izsvenezie.it (A.S.); asalomoni@izsvenezie.it (A.S.); smaniero@izsvenezie.it (S.M.); izambon@izsvenezie.it (I.Z.); fbonfante@izsvenezie.it (F.B.); imonne@izsvenezie.it (I.M.); abortolami@izsvenezie.it (A.B.); 3 Clinical and Applied Virology Group, Department of Infection and Immunity, Luxembourg Institute of Health, 29, Rue Henri Koch, Esch-sur-Alzette, L-4354 Luxembourg, Luxembourg; chantal.snoeck@lih.lu; 4 Faculty of Veterinary Medicine–Skopje, Ss. Cyril and Methodius University in Skopje, Lazar Pop Trajkov 5-7, 1000 Skopje, North Macedonia; adodovski@fvm.ukim.edu.mk; 5 Avian Virology and Immunology, Sciensano, Rue Groeselenberg 99, 1180 Ukkel, Belgium; steven.vanborm@sciensano.be (S.V.B.); mieke.steensels@sciensano.be (M.S.); 6 Section Veterinary Services (1417), Laboratory for Animal Health Virology, 79, Athalassa Avenue, Aglantzia, Nicosia 2109, Cyprus; vchristodoulou@vs.moa.gov.cy; 7 Institute For Diagnosis and Animal Health, 63, Dr. Staicovici Str., Sector 5, 050557 Bucharest, Romania; iuliana.onita@idah.ro (I.O.); raluca.burlacu@idah.ro (R.B.); 8 Laboratorio Central de Veterinaria (LCV), Ministry of Agriculture, Fisheries and Food, Ctra. M-106, Km 1, 4 Algete, 28110 Madrid, Spain; azusan@mapa.es; 9 National Reference Laboratory for Avian Influenza and Newcastle Disease, Federal Centre for Animal Health (FGBI “ARRIAH”), Vladimir 600901, Russia; chvala@arriah.ru; 10 National Veterinary Services Laboratories, U.S. Department of Agriculture, Ames, IA 50011, USA; mia.kim.torchetti@usda.gov; 11 National Veterinary Research Institute, Vom 93010, Nigeria; ismaila.shittu@gmail.com (I.S.); mayowaolabode12@gmail.com (M.O.)

**Keywords:** NDV, machine learning, sequencing, cross-protection, hemagglutination inhibition, antigenic cartography

## Abstract

Newcastle disease virus (NDV) continues to present a significant challenge for vaccination due to its rapid evolution and the emergence of new variants. Although molecular and sequence data are now quickly and inexpensively produced, genetic distance rarely serves as a good proxy for cross-protection, while experimental studies to assess antigenic differences are time consuming and resource intensive. In response to these challenges, this study explores and compares several machine learning (ML) methods to predict the antigenic distance between NDV strains as determined by hemagglutination-inhibition (HI) assays. By analyzing F and HN gene sequences alongside corresponding amino acid features, we developed predictive models aimed at estimating antigenic distances. Among the models evaluated, the random forest (RF) approach outperformed traditional linear models, achieving a predictive accuracy with an R^2^ value of 0.723 compared to only 0.051 for linear models based on genetic distance alone. This significant improvement demonstrates the usefulness of applying flexible ML approaches as a rapid and reliable tool for vaccine selection, minimizing the need for labor-intensive experimental trials. Moreover, the flexibility of this ML framework holds promise for application to other infectious diseases in both animals and humans, particularly in scenarios where rapid response and ethical constraints limit conventional experimental approaches.

## 1. Introduction

Vaccine discovery has been described as “One of the brightest chapters in the history of science”, due to its impact on human and animal health [1,2]. Vaccination has successfully eradicated smallpox and rinderpest and it has provided effective control for several decades against several human and animal diseases like measles, pertussis, diphtheria, mumps, rabies, and foot and mouth disease [3,4,5]. Nevertheless, emerging or remerging pathogens and continuous viral evolution represent a challenge for modern vaccinology [6,7]. One of the major obstacles in vaccine development and disease control is the extreme variability of some of these pathogens, in particular, RNA viruses [8]. Many viruses are characterized by limited cross-protection among different strains of the same species and, even if the development of universal vaccines would probably be the optimal solution [9], this goal is far from being achieved for most diseases. The current approach is to update or validate new vaccines to respond to new viruses or genotypes [10]. Unfortunately, this approach is time consuming and expensive, limiting the prompt application of control strategies or discouraging it when economic benefits are not significant. Additionally, when several vaccines are already available for a given disease, the choice of the best one is typically due to personal opinion rather than facts, mainly as a consequence of high costs or ethical constraints in performing adequate experimental testing [11,12,13,14].

Newcastle disease, one of the most important avian diseases worldwide, is caused by *Orthoavulavirus javaense* (https://ictv.global/taxonomy; accessed on 26 March 2024), historically and commonly known as Newcastle disease virus (NDV), an enveloped, single-stranded RNA virus member of the genus *Orthoavulavirus* from the *Paramyxoviridae* family [15,16].

Similarly to other RNA viruses, NDV features a high evolutionary rate [17]. Currently, two classes—class I and class II—20 genotypes, and multiple subgenotypes have been defined based on the Fusion (F) gene sequence analysis [18]. Such genotypic heterogenicity reflects on relevant biological implications, including virulence. Clinical signs in infected birds are extremely variable, from subclinical to fatal, depending on host and virus-related factors. NDV strains are often also classified according to their virulence with lentogenic NDV strains causing subclinical infections with mild respiratory or enteric disease. Mesogenic NDV strains have intermediate virulence and cause respiratory infections with a moderate mortality rate while velogenic (virulent) strains are further divided into two types: viscerotropic velogenic strains and neurotropic velogenic strains. The former can cause lesions mainly affecting the intestinal tract such as ulcerative hemorrhages in the mucosa; lymphoid depletion; and necrotic foci in the spleen, liver, and gut-associated lymphoid tissue (GALT). Neurotropic velogenic strains are characterized by lesions affecting primarily the CNS, which results in dyspnea, depression, opisthotonos, head twisting, and paralysis [15,19].

Because of clinical and economic relevance, NDV vaccination has a long history, being first proposed in the early 1930s and used extensively since then, becoming one of the most applied vaccines in veterinary medicine [20]. Several vaccines, both live and inactivated, based on avirulent, lentogenic, and mesogenic strains have been developed over time and more recent advances in molecular biology have further widened the portfolio to recombinant vaccines [21,22]. Nevertheless, NDV strains included in commercially available conventional vaccines, which still represent the most common choice in many countries, belong to genotypes I (Ulster, QV4, VG/GA-AVINEW) and II (LaSota, B1, VG/GA), and thus are phylogenetically divergent from strains circulating in the last two decades in endemic Middle Eastern and Asian countries, where genotype VII is the most prevalent [23,24]. Newer commercially available recombinant vaccines have been developed by using the turkey herpesvirus as a vector for the expression of NDV F and or HN proteins through reverse genetics and recombination technologies. However, for some of these newer vaccines, in terms of match towards the field viruses, this did not represent a major advancement as the genetic material was derived from old vaccine strains (e.g., LaSota clone 30) rather than from field velogenic viruses [25]. However, HVT-vectored vaccines present many favorable characteristics such as easy production, favorable characteristics for mass administration in hatcheries, robust stimulation of cell-mediated immunity, less interference from maternally derived antibodies, and more [26]. Despite the wide use of these vaccines, NDV still represents a major menace for the worldwide poultry industry, and the development of novel NDV vaccines, which can induce better protection with safer characteristics, is ongoing [21,22]. Protection against clinical disease by commercially available vaccines has been demonstrated in several experimental trials, while protection from shedding has been less investigated and on some occasions was found to be poorly related to protection from disease [27,28]. Moreover, vaccine administration in the field is often suboptimal and provided according to a plethora of poorly standardized protocols, which further hinders vaccine-induced protection [21]. The circulation of virulent NDV in vaccinated poultry has been reported [29,30], highlighting the need for an improved understanding of the protection offered by current vaccines and the importance of accurate selection of immunization strategies to reduce the silent circulation of virulent NDV.

Despite NDV being considered a single serotype, several reports and studies have highlighted the effect of virus genetic variability on antigenic features and thus on cross-protection. Antibodies raised against the transmembrane hemagglutinin-neuraminidase (HN) and fusion (F) proteins are regarded as neutralizing, being able to block viral attachment and fusion, respectively [22,31,32,33,34]. The immune response is commonly monitored through a hemagglutination inhibition (HI) test that, although not fully representative of protection and viral shedding inhibition, is nevertheless of remarkable help to study cross-protection, particularly when applied in the framework of more modern techniques like antigenic cartography. Antigenic cartography was initially developed to measure the antigenic diversity between influenza viruses by comparing the reaction titers among the test antigens and reference antisera [35]. Its use has now been extended to other important pathogens to understand antigenic relatedness and to guide vaccine strain selection as antigenic cartography can simplify data interpretation through intuitive antigenic maps [35].

Unfortunately, such in vitro studies are laboratory intensive, requiring dedicated structure, skilled personnel, and availability of standardized and well-characterized viral strains and sera, which might not be available in several socio-economic contexts. Moreover, such methods do not have the flexibility and rapid turnaround time necessary to deal with farming routines and with a heterogeneous and/or rapidly evolving epidemiological scenario.

For this reason, different machine learning approaches have been evaluated in the present study to predict the antigenic distance among NDV strains based on the comparison of their genetic sequences. NDV has been chosen as a model for different reasons: (1) It is one of the most economically relevant diseases of poultry, which has also prompted relevant research over the years. (2) Like other RNA viruses it is characterized by a remarkable genetic variability. (3) The major targets of the host immune response, responsible for the viral neutralization, are well characterized and commonly sequenced. (4) The HI test for NDV is commonly applied in several diagnostic and research laboratories for the estimation of vaccine coverage in vaccinating countries and for surveillance purposes in non-vaccinating countries. (5) Different vaccines based on major, but not all, genotypes have been developed. Nevertheless, the choice of the best vaccine according to the field epidemiological scenario is gaining interest among public authorities and veterinarians [36,37].

The current study aims to provide a new approach to predict antigenic relationships between different strains based on an inexpensive, rapid, and objective approach, which could lead to an improved planning of adequate control strategies, not only for NDV but also for other human and animal diseases.

## 2. Materials and Methods

### 2.1. Viruses

Viruses from the repository of the European Reference Laboratory (EURL) for Avian Influenza and Newcastle Disease were selected to be representative of the current NDV epidemiological situation and of vaccine seed strains. The selection of strains voluntarily overrepresented genotypes more frequently associated with outbreaks in domestic poultry and was limited by the availability of viral strains. All viruses were propagated and titrated in 9–10-day-old embryonated SPF chicken eggs via the chorioallantoic sac route. The median embryo infectious dose (EID_50_) was calculated using the standard method as previously described [38]. A list of selected viruses is available in Table 1.

Harvested allantoic fluids were confirmed for NDV by hemagglutination (HA) and hemagglutination-inhibition (HI) assays using NDV-specific antiserum (Ulster 2C strain) according to standardized protocols available at the EURL website (https://www.izsvenezie.com/reference-laboratories/avian-influenza-newcastle-disease/diagnostic-protocols/, accessed on 1 March 2025). The virus-containing allantoic fluids were harvested and stored at −80 °C until use.

### 2.2. Preparation of Hyperimmune Sera in SPF Chickens

To obtain the chicken hyperimmune sera, 3000 HAU of each selected strain (Table 1) was inactivated with formalin at 37 °C at a final concentration of 0.1% for 18 h and inoculated into three 6-week-old SPF chickens through an intravenous route [40]. The chickens were boosted with the same amount of inactivated virus after three weeks from the first immunization. The animals were bled on day 21 after the second immunization and the sera of the three immunized birds were pooled to obtain a representative serum and reduce the effect of biological variability in immune responses. Sera were subjected to complement inactivation by heating at 56 °C for 30 min, aliquoted in small volumes, and stored at −20 °C until use.

A second immunization experiment was performed immunizing 5 SPF chickens with either NDV/chicken/rus/Krasnodar/91/19, APMV-1/avian/Nigeria/21RS2368-1/2021, or LaSota antigens as described above. Following bleeding at 21 days after the second immunization, individual sera of each immunized bird were collected, subjected to complement inactivation by heating at 56 °C for 30 min, aliquoted in small volumes, and stored at −20 °C until use.

Immunizations were carried out in accordance with the Italian and EU law on Animal Welfare (Italian Legislative Decree No. 26 dated 4th March 2014 implementing the European Directive 2010/63/EU) and were approved by IZSVe’ Ethics Committee (Organismo Preposto al Benessere animale).

### 2.3. Haemagglutination Inhibition Assay

Haemagglutination inhibition assays were performed according to standard procedures using four HA units of antigen per well (https://www.woah.org/fileadmin/Home/eng/Health_standards/tahm/A_summry.htm, accessed on 30 December 2024). Back-titration of each antigen used was performed to confirm that four HA units per well were present. The test results were accepted if the control sera were within a two-fold dilution range of their known HI titer. The HI titers were read as the reciprocal of the highest dilution showing complete inhibition. The HI titers were expressed as the reciprocal of log2 in this study. Sera with HI titers ≥4 were considered positive according to the WOAH criteria. All tests were repeated three times by two different technicians.

### 2.4. Microneutralization Assay

The serum-neutralizing antibody titers were detected with a microneutralization (MN) assay using chicken embryo fibroblasts (CEF) of SPF 11-day-old chicken embryos prepared as previously described [41]. CEF cells were seeded in 96-well plates and used for virus neutralization tests after 24 h at full confluency. Serum samples were then serially diluted two-fold from 1:10 to 1:1280 in Dulbecco’s Modified Eagle Medium (DMEM) and incubated with an equal volume of virus at a final concentration of 100 TCID_50_/100 µL for 1 h at 37 °C. One hundred microliters of the virus-serum mixture were then used to infect the cell monolayers. After 1 h of incubation, 50 µL of DMEM was added and the plate was incubated at 37 °C. The presence of NDV in infected cells was detected by ELISA after 72 h of incubation as previously described, with minor modifications [42]. Briefly, plates were fixed with a cold fixative solution (80% acetone) for 10 min at room temperature. After removal of the fixative, the plates were allowed to dry and washed 3 times with PBS. A primary monoclonal antibody against NDV (Mouse Newcastle Disease Virus (NDV) Monoclonal Antibody MBS312296, MyBiosource, Inc, San Diego, Southern California, USA)) was added to each well and incubated for 1 h at room temperature. Plates were washed three times and a secondary HRP-conjugated antibody (Peroxidase AffiniPure™ Goat Anti-Mouse IgG (H + L), Jackson ImmunoResearch Europe Ltd.) was added to each well. The plate was then incubated for 1 h at room temperature. After removal of the secondary antibody and washing, 100 µL of a freshly prepared substrate (10 mg OPD, Sigma-Aldrich in 20 mL citrate buffer + H_2_O_2_) was added to each well. Absorbance was read at 490 nm (OD490) using a spectrophotometer (Tecan Trading AG, Männedorf, Switzerland), and the virus neutralization antibody 50% titer of each serum was calculated using the following equation:

x = ((average OD of Virus Control wells) − (average OD of Cell Control wells))/2

All values below or equal to x have been considered positive for neutralization activity.

### 2.5. Antigenic Cartography

The antigenic variation among the different NDV strains was quantified and visualized using the antigenic cartography method [43] with 1000 bootstrap replicates. Briefly, the target distance between an antiserum A and an antigen B was determined by calculating the difference between the maximum logarithm (log2) reciprocal HI or MN titer for antiserum A against any antigen and the log2 reciprocal neutralizing titer for antiserum A against antigen B. The distance obtained is expressed as antigenic units (AU) with one AU corresponding to a 2-fold change in the titer. Using the multidimensional scaling method [44], the position of each virus and antiserum in the map will be the result of minimizing the difference between the target distances and map distances. The distance between the points on the 2D map represents the antigenic distances.

### 2.6. Sequencing and Phylogenetic Analysis

RNA was purified using QIAamp Viral RNA Mini Kit (Qiagen, Hilden; Germany) following the manufacturer’s instructions. Sequencing libraries were obtained using the Nextera XT DNA Sample Preparation Kit (Illumina; San Diego, California, USA) starting from amplification products obtained using SuperScript™ III One-Step RT-PCR System with Platinum™ Taq High Fidelity DNA Polymerase kit (Thermo Fisher Scientific; Waltham, Massachusetts, USA) (primer sequences available upon requests) or from double-stranded cDNA generated using Maxima H Minus Double-Stranded cDNA Synthesis (Thermo Scientific™). Libraries were quantified using the Qubit dsDNA High Sensitivity Kit (Invitrogen, Waltham, Massachusetts, USA). The indexed libraries were pooled in equimolar concentrations and sequenced on the Illumina MiSeq platform. Reads were clipped from Illumina Nextera XT adaptors using scythe v0.991 (https://github.com/vsbuffalo/scythe) (accessed 12 December 2022) and trimmed with sickle v1.33 (https://github.com/najoshi/sickle) (accessed 12 December 2022). Reads shorter than 80 bases or unpaired after previous filters were discarded. High-quality reads were aligned against a reference genome using BWA v0.7.12. Alignments were processed with Picard tools v2.1.0 (http://picard.sourceforge.net, accessed on 1 March 2025) and GATK v3.530-32 to correct potential errors, realign reads around indels, and recalibrate base quality. Single nucleotide polymorphisms (SNPs) were called using LoFreq v2.1.233, and the outputs were used to generate consensus sequences. Consensus sequences of the complete genomes were submitted to GenBank under accession numbers (PV137993–PV138014). The obtained F sequences were used to evaluate the relationship among the considered strains and classify them. To this purpose, a reference dataset of 128 sequences representing all class II NDV genotypes and sub-genotypes (available at https://github.com/NDVconsortium/NDV_Sequence_Datasets/tree/master) (accessed 1 March 2023) [18] was downloaded and aligned with the sequences obtained in the present study. A maximum likelihood phylogenetic tree was reconstructed using IQ-Tree, selecting as the substitution model the one with the lowest Akaike information criterion (AIC), calculated using the same software. The reliability of the inferred clades was assessed by performing 1000 bootstrap replicates.

### 2.7. Database Preparation

All generated HN sequences were aligned at the codon level using the MAFFT method [45] implemented in TransaltorX [46]. A database was constructed, reporting the following for each strain pair: (1) the antigenic distance (dependent variable), (2) the genetic distance between strains, and (3) a site-by-site comparison between amino acids features. Amino acids can be grouped according to their physical–chemical features and those can significantly affect the protein structure as well as its immunogenicity. To include these features in the model, the metric described by Atchely et al. was used [47]. Briefly, the main features of each amino acid were described as a combination of 5 continuous variables (polarity index, secondary structure factor, volume, Refractivity/Heat Capacity, and Charge/Iso-electric point) obtained through factorial analysis and representative of the variability described originally by more than 400 chemical–physical properties [47]. The absolute values of the difference between vaccine and challenge strain were then calculated position by position for all the five factors and included in the database. Positions without variability were removed from the database. To reduce the curse of dimensionality effect, variables with a coefficient of correlation higher than 0.7 were removed from the dataset.

Before algorithm development and optimization, 40% of the records were randomly selected, removed, and stored as the test dataset. The following steps were thus performed on the remaining data (i.e., training dataset).

The same approach was performed on the obtained F sequences and on the concatenation of both the HN and F sequences dataset (renamed Merged dataset). The ML models were developed and validated on all the defined datasets.

### 2.8. Protection Prediction

In most of the literature, linear models using the genetic distance between strains as a predictive variable have been considered [48] and were thus herein used as a baseline model. However, many potentially important variables are neglected using this approach. On the other end, data mining methods, such as machine learning-based approaches, are capable of providing an effective way of overcoming these limitations by analyzing large sets of predictive variables and modeling complex, potentially non-linear relationships.

Keeping this in mind, the following approaches were implemented and trained on the datasets described in Section 2.7: Bagging trees (BT), Random forest (RF), Artificial Neural Network (ANN), and Support Vector Machines (SVM). The following R [49] libraries were used for analysis and data visualization: *ape* [50], *seqinr* [51], *ips* [52], *ggplot2* [53], *lattice* [54], *caret* [55], *randomForest* [56], *neuralnet* [57], *kernlab* [58], and *mboost* [58].

#### 2.8.1. Bagging Trees (BT)

Those methods belong to the class of tree-based methods, which involve the stratification of predictor space in many simpler regions; the tree-like structure trained in this process can be then used for prediction purposes. The bagging method uses a bootstrap approach to deal with the high variance that typically affects regression trees. Briefly, several bootstrapped datasets are generated by random sampling with replacement. For each dataset, a regression tree is trained and the final prediction is obtained through averaging of the different models [59].

#### 2.8.2. Random Forest (RF)

RF methods use an approach similar to bagging trees; different decision trees are built on bootstrapped datasets. However, each time a split in the tree is considered, only a random subset of *n* predictors is selected as the split candidate. This approach allows the bootstrapped trees to decorrelate, making the average results less variable and consequently more accurate. Additionally, using only a random subset of all the features, random forest can handle big datasets, efficiently dealing with the “course of dimensionality” [60,61,62].

#### 2.8.3. Artificial Neural Network (ANN)

ANNs are models inspired by our understanding of biological brain behavior. A typical neural network is defined by one or more layers of neurons, called nodes (i.e., input nodes, hidden nodes, and output nodes), connected among them by “axons” and “synapses”. Each node integrates the information received from the database or previous layers through an activation function determining the output of that node, which is transferred to the following one with a certain weight (w). As in biological systems, synapses can discharge with a different strength, manipulating the data in the calculations. Weights, and consequently neural network prediction, are typically trained using a backpropagation approach, evaluating iteratively the prediction errors and defining the best set of weights that reduce the total error of the network. To summarize, an ANN can be defined by the activation function, the network topology (i.e., number of layers and nodes for each layer) and the training algorithm.

#### 2.8.4. Supporting Vector Machines (SVM)

SVM is a generalization of the maximal margin classifier that can be used to model both nominal (classification) and quantitative (regression) problems. A key feature of SVMs is their ability to map the data into a higher dimensional space using the so-called “kernel trick”. Non-linear kernel functions can be used to transform the original data (e.g., highly complex non-linear relationships) to a new high dimensional feature space where the input data become more separable compared to the original feature space by causing the non-linear relationship to appear linear in the new feature space. In other words, the kernel trick involves a step in which new features, expressing mathematical relationships between measured variables, are added, allowing the SVM to learn concepts that were not evident in the original dataset.

### 2.9. Performance Criteria

The performances of each method were evaluated through the coefficient of determination (R^2^), mean absolute error (MAE), and root-mean square error (RMSE), calculated using the difference between the predicted value (Ft) and the actual one (Yt).

The MAE is thus an arithmetic average of the absolute errors.

$$MAE=\frac{\sum_{t=1}^{n}\left|Yt-Ft\right|}{N}$$

The RMSE corresponds to the sample standard deviation of the differences between predicted values and observed values:

$$RMSE=\sqrt{\frac{\sum_{t=1}^{n}{\left(Yt-Ft\right)}^{2}}{n}}$$

Consequently, the smaller the value the better the model performed.

On the contrary, the R^2^ is an index of how well the model fits the data.

$$R^{2}=1-\frac{\sum_{t=1}^{n}{\left(Ft-Yt\right)}^{2}}{\sum_{t=1}^{n}{\left(Yt-\bar{Y}\right)}^{2}}$$

Unlike the RMSE, the higher the R^2^ (0 < R^2^ < 1), the better the performance for the compared models. The metrics were chosen for their complementary strengths—R^2^ to measure variance explained and overall fit, RMSE to penalize large errors, and MAE to provide a robust average error—thus ensuring a balanced evaluation of the model’s predictive performance.

### 2.10. K-Fold Cross-Validation

All tested methods allow different settings to modify their complexity and flexibility. While increasing the model flexibility improves its fit (i.e., lower bias) to the data and its predictive performance on the training dataset, this can lead to an excessive specificity for this dataset, making the prediction useless when applied to an unknown dataset (i.e., higher variance), a phenomenon called overfitting.

To minimize the trade-off between bias and variance, each method was optimized through a repeated cross-validation approach (5-fold cross-validation approach repeated 20 times).

In k-fold cross-validation, the original dataset (D) is randomly split into k mutually exclusive subsets (the folds: D1, D2, …, Dk). The prediction model is then trained and tested k times. All but one fold are used for training while the remaining is used as a test dataset. The whole process is then repeated t times. The cross-validation estimate of the overall performance criteria was consequently calculated as the average of the k × t individual performance:

$$CV=\frac{1}{k\times t}\sum_{j=1}^{t}\sum_{i=1}^{k}{RMSE}_{ij}^{2}$$

where CV stands for cross-validation, k is the number of folds used, t is the number of repetitions, and RMSE is the performance measure used in this study.

The settings optimized for each method are reported in Supplementary Table S1.

For each method, the model displaying the best mean RMSE calculated on the test dataset was selected as the final model.

### 2.11. Model Comparison

All R^2^, MAE, and RMSE values sampled for each final model were then used to evaluate the presence of statistically significant differences among methods in predicting the protection outcome. A pairwise *t*-test with Bonferroni correction was used for this purpose setting the statistical significance to *p*-value < 0.05.

### 2.12. Best Model Evaluation on the Test Dataset

The final assessment of the developed model performances was conducted using the test dataset, which was never included in any step of the validation process and can thus be considered fully independent (i.e., representative of potential new data generated in real-life scenarios). The antigenic distance among strain pairs was predicted using the best model based on the independent variables of the test dataset and compared to the real values obtained experimentally. The predictive performance of a linear model (LM) considering genetic distance only was also evaluated for comparison purposes.

## 3. Results

### 3.1. Genetic and Antigenic Relatedness by Antigenic Cartography

The phylogenetic analysis and classification demonstrated that the selected strains belong to 13 genotypes (Supplementary Figure S1). Specifically, the field strains belong to genotypes IV, V.1, VI.2.1, VII.1.1, VII.2, XIV.2, XVII, XXI.1.1, and XXI.2, which are representative of most of the virulent genotypes responsible for important recent outbreaks in Europe and West Africa, while the vaccine strains belong to genotypes I.1.1, I.1.2, I.2, and II (Table 1).

The average raw nucleotide distance among obtained sequences was 13.2% [interval = 0–19.3%] and 14.0% [interval = 0–19.1%] at F and HN gene levels, respectively.

Compared to the vaccine strains, the field strains present from 28 to 79 and from 34 to 84 amino acid differences in the F and HN proteins, respectively (Table 2).

**Figure 1:**
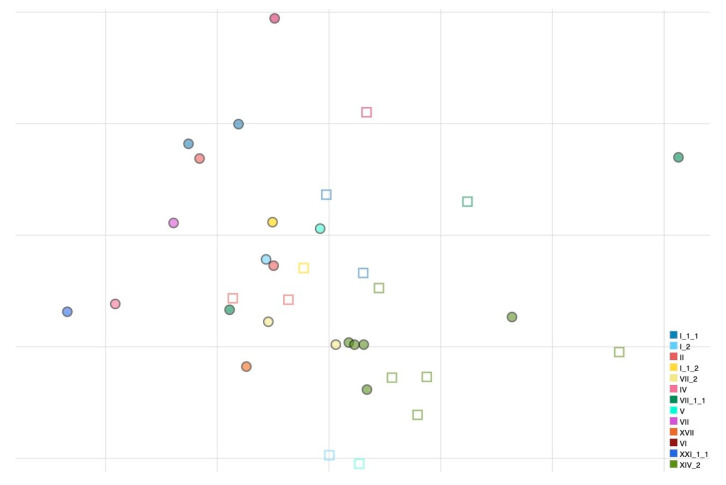
Antigenic map of NDVs based on HI data. Names of antigens (depicted as dots) and sera (depicted as squares) were excluded from the map to improve readability. Colors have been assigned to each genotype (nomenclature according to Dimitrov et al., [18]) to visualize antigenic relatedness between genotypes. The vertical and horizontal axes both represent antigenic distance, and, because only the relative positions of antigens and antisera can be determined, the orientation of the map within these axes is free. The spacing between grid lines is 1 unit of antigenic distance corresponding to a twofold dilution of antiserum in the HI assay.

Antigenic cartography was used to characterize the antigenic relatedness among all viruses used in this work using the HI values obtained (Figure 1). A prediction test was carried out to evaluate the reliability of the cartography and the optimal number of dimensions to represent the dataset. The final antigenic map was represented in 2D as no discernible mean advantage in precision was obtained using higher dimensions.

All genotype I and II strains that are used for vaccine formulations appear to be closely related antigenically (AU distances ranging from 0.097 to 1.271). Genotype I can be distinguished from genotype II strains by 28 and 20 amino acid substitutions in F and HN proteins, respectively. Differently, large distances in AU from vaccine strains were observed for some virulent strains belonging to genotypes VII.1.1 and XIV.2 (e.g., APMV-1/chicken/rus/Krasnodar/91_21VIR4521/19 and APMV-1/avian/Nigeria/21RS2368-1/2021, respectively). APMV-1/chicken/rus/Krasnodar/91_21VIR4521/19 presents 60–74 amino acid differences from the vaccine strains in the F gene and 56–69 differences in the HN protein, while APMV-1/avian/Nigeria/21RS2368-1/2021 presents 59–76 amino acid differences from the vaccine strains in the F protein and 66–79 differences in the HN protein (Table 2). Within genotypes, antigenic diversity was observed inside genotype XIV.2, a genotype for which multiple isolates were available due to the current endemic situation in Nigeria. Genotype XIV.2 viruses have 10–30 amino acid differences at the HN gene level and 3–14 at the F gene level, highlighting a considerable diversity given that they have been collected during a short time interval and in the same geographical region. In addition, one isolate (APMV-1/avian/Nigeria/21RS2368-1/2021) is antigenically distinct (0.926–1.241 AU) from other genotype XIV.2 isolates.

PPMV-1 viruses (APMV-1/pigeon/Italy/19VIR8321/2019 and APMV-1/pigeon/Luxembourg/18175752_18VIR10959/2018) grouped together in the antigenic map and despite not being antigenically far from vaccine strains, they were distinguishable from other NDV strains.

**Figure 2:**
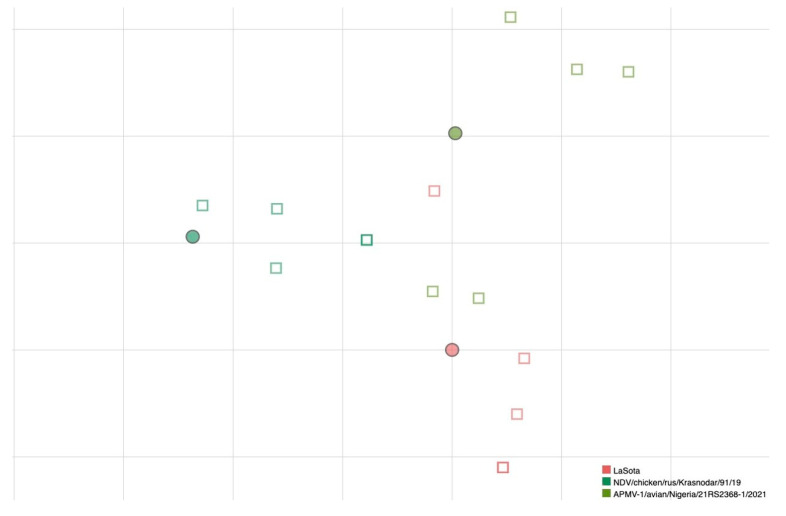
Antigenic map of NDVs based on MN data. Dots represent antigens and squares represent sera of individual immunized birds. The same color has been used for viruses and homologous antisera; superposition of two sera is represented by darker color of the square. The spacing between grid lines is 1 unit of antigenic distance corresponding to a two-fold dilution of antiserum in the MN assay.

MN data obtained from a further selection of antigens, based on the antigenic map created based on HI results, were used to test if antigenic relatedness obtained by HI also reflected the neutralizing ability of sera. A larger number of replicates has been used to further strengthen the reliability of results for this smaller selection of viruses. Results are presented in Figure 2, where a grouping of sera with the homologous antigen is visible.

### 3.2. Dataset and Algorithms Development and Validation

After dataset elaboration and pre-processing, a total of 190 strain comparisons were considered and 68, 89, and 120 independent predictive variables were retained in the final F, HN, and Merged datasets, respectively.

**Figure 3:**
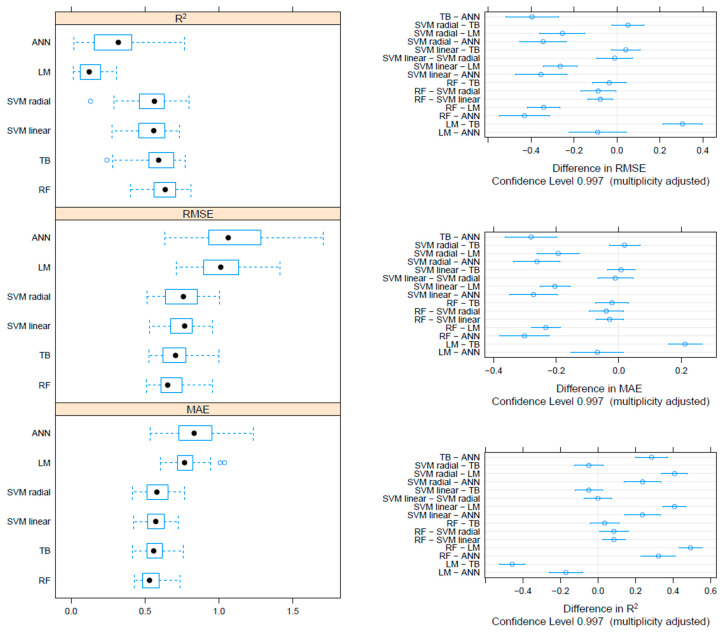
Boxplot performance metrics obtained through cross-validation for different methods, based on the F dataset (**left**). The solid, hollow dots, represent the median value. Differences in performance parameters between methods pairs (**right**). The average difference and the confidence interval, corrected for multiple comparisons, indicative of statistical significance, are reported.

**Figure 4:**
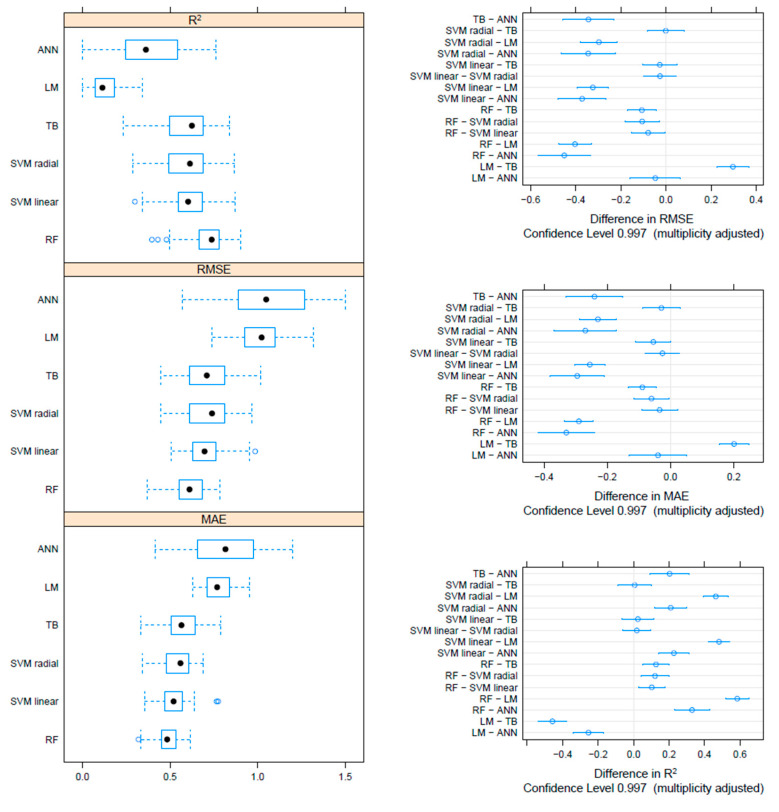
Boxplot performance metrics obtained through cross-validation for different methods, based on the HN dataset (**left**). The solid, hollow dots, represent the median value. Differences in performance parameters between methods pairs (**right**). The average difference and the confidence interval, corrected for multiple comparisons, indicative of statistical significance, are reported.

**Figure 5:**
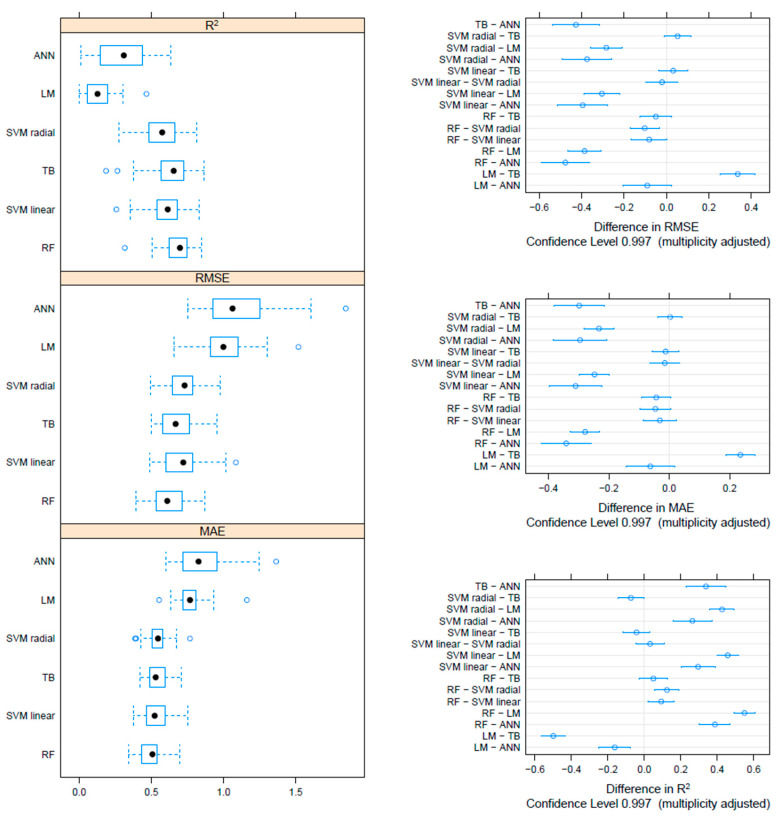
Boxplot performance metrics obtained through cross-validation for different methods, based on the Merged dataset (**left**). The solid, hollow dots, represent the median value. Differences in performance parameters between methods pairs (**right**). The average difference and the confidence interval, corrected for multiple comparisons, indicative of statistical significance, are reported.

On the F gene-based dataset, the metrics collected through repeated cross-validation showed overall better performances of the tree-based methods (TB and RF) compared to SVM, although the difference was not statistically significant. On the other hand, both tree-based and SVM approaches significantly outperformed ANN and linear models (Figure 3). Essentially comparable results were obtained on the HN dataset, although in this case, the RF model metrics were typically significantly better than the other approaches, including TB and SVM (Figure 4). An intermediate pattern featured the Merged dataset, with RF outperforming the other approaches except for TB, against which the improvement was only marginally significant (Figure 5).

When the three datasets were compared, RF methods developed on the HN and Merged dataset showed overall better performances, while RF based on the F gene performed comparably to the SVM and TB methods (Supplementary Figure S2). The differences were not statistically significant except for the HN-based RF approach, whose performances were significantly better than most of the other approaches independently from the considered metric (Supplementary Figure S3). Regardless of the dataset, the ANN and LM showed significantly worse performances.

### 3.3. Final Model Testing on the Test Dataset

Based on the overall observed performance patterns, the HN-based RF method was selected and its predictive performances were assessed using the test dataset. The LM was also evaluated on the same dataset for comparison purposes. LM metrics were RMSE = 0.934, MAE = 0.753, and R^2^ = 0.051, while the RF were RMSE = 0.509, MAE = 0.382, and R^2^ = 0.723.

## 4. Discussion

Machine learning (ML) was, from the very beginning, applied to medical datasets [63]. The growing availability of new algorithms, big data, and computational power has led to its wide use as an aid in physicians’ decision-making processes [63]. ML has been applied to disease classification, intervention outcome, and survival prediction, with different applications in oncology, transplantology, cardiology, and diagnostic imaging, just to mention a few [64,65,66,67,68]. Infectious diseases and vaccinology have also been involved in this revolution [69,70].

The relationship between genetic distance and cross-protection is typically recognized and has a strong biological and evolutive background. Nevertheless, this correlation has been proven poor, and many exceptions have been reported. This is not unexpected considering the non-direct relationship between genotype and phenotype and the different weights of protein regions and domains from an immunological point of view. Substitutions involving different positions and/or amino acids with diverse chemical–physical properties can severely affect the cross-protection among strains. In fact, while genetic distances correlate with the amino acid ones [34], the differences in specific protein sites can be large, and a simple sequence comparison might not be effective in predicting antigenic similarities. This evidence explains the different and sometimes unexpected findings observed in experimental cross-protection studies involving different genotypes and suggests that the selection of vaccine antigens should still be based on dedicated experimental studies with live animals [33,71].

However, in vivo challenge studies are expensive, available only in a limited number of laboratories with dedicated facilities, and are extremely time consuming, making them poorly suited to the requirements of modern farming systems, dealing with a dynamic and rapidly changing epidemiological scenario.

NDV was not an exception since different studies have demonstrated a differential cross-protection induced by homologous versus heterologous challenges after previous immunization [33,36,72]. The antigenicity of genotypes and strains can be differentiated by cross-HI assays, which correlate with vaccine protection measured by virus shedding after challenge, and a certain linear relationship was observed between antibody titer and viral shedding [34]. The choice of the best vaccine should be based on the knowledge of the viral features of strains circulating in each country. For example, the value of the calculation of antigenic distances for vaccine selection is well demonstrated by the process of selection of vaccines for seasonal human influenza. In the process of selection of vaccine strains, the antigenic profiles of circulating strains are compared with those of existing vaccine strains and if the antigenic distance (difference) between a vaccine strain and circulating strains is significant, it may warrant an update of the vaccine [73].

The ML methods herein developed aim to overcome these limitations by validating more informed and flexible antigenic distance predictive algorithms. Including site-specific amino acid features in our model, as well as using methods able to model non-linear relationships, allowed us to significantly improve antigenic distance prediction compared to linear models and, at the same time, allow for a much quicker response time.

Different methods behaved differently, emphasizing the need to carefully evaluate different approaches and validate them to set the best model for each dataset. The random forest approach granted the best performances, significantly outperforming linear models and most of the other ML methods. In particular, the RF trained on the HN dataset led to the lowest estimate error and better R^2^, although not significantly better than the F gene-validated approach.

While the good performance obtained using the F dataset might seem surprising, the HI test being biologically grounded on the neutralization of the HN-mediated attachment and being affected only to a limited extent by the presence of neutralizing antibodies directed towards epitopes located on the F protein [74], the prediction capability of F sequences was largely expected because of the common evolutive history of the two genomic segments within each virus, making the respective amino acid profiles highly correlated. This statement is supported by the lack of any predictive improvement when the merged HN and F dataset was used, which, on the other hand, outperformed the F-based dataset alone. Such evidence testifies that the variability depicted by the HN protein overlaps the F one, and no residual information is provided by this gene.

To understand how the antigenic differences determined by HI were related to the neutralizing ability of sera against live NDV viruses, a smaller panel of viruses, selected on the basis of HI results among the ones where the greatest differences were identified, were tested by MN with five homologous antisera generated specifically for this purpose. The number of strains selected for cross-neutralization experiments represents a limitation of the study and MN assays have been used to strengthen and verify the robustness of antigenic data obtained by HI. It has been demonstrated that simultaneous neutralization of different epitopes on NDV viruses is required to fully neutralize the infectivity of the virus, and therefore it has been postulated that serum neutralization assays could be a better predictor of the protective ability of candidate vaccine strains [36]. In our study, antigenic distances identified by MN were similar to those obtained by the HI for the three tested viruses, even accounting for some variability observed between individual replicates (i.e., biological variability).

In this framework, it must be stressed that the developed algorithms are not intended to identify any biologically relevant feature of the considered viruses (e.g., neutralizing antigens, specific domains, glycosylation sites, etc.) but simply to predict an outcome of interest (i.e., antigenic distance) based on a set of variable (i.e., gene sequences). For the same reason, any a priori knowledge of NDV biology was voluntarily ignored. This decision was supported by two main reasons. At first, the variability of the results of experimental trials, ascribable to experimental procedures, laboratory techniques, animal features, etc., while fundamental to gaining an overall qualitative understanding of pathogen biology, lack the necessary rigor to be included as predictive variables in quantitative mathematical models without setting subjective and arbitrary inclusion criteria. For the same reason, their use would hamper the future expansion and update of the algorithm training database, necessary to continuously improve its performance, since experimental procedures would be required, followed by subjective data elaboration. The use of sequence data allows not only a much faster and cheaper data generation but also dramatically reduces future problems of data standardization among laboratories.

Despite the encouraging results, a strong focus should be maintained on the limitations of this study, which are primarily related to the reduced availability of experimental data. The number of strain combinations tested in experimental trials made it difficult to extensively train the different models and to confidently test their performances. Even if an extensive cross-validation approach was used, a certain dependency between the model selection and the training dataset could not be avoided being the final model selected, optimized, and validated iterating over partitions of the training dataset. However, the R^2^ of the linear model estimated using traditional parametric assumption was lower but comparable (i.e., 0.110) with that calculated using cross-validation (i.e., 0.128), suggesting that although a certain overfit is possible and could have also affected the other approaches’ results, its effect should be minimal, supporting the reliability of the results. Moreover, the use of a proper test dataset, not involved in the optimization and validation process, led to metrics estimations comparable with the ones obtained through cross-validation (e.g., average RF R^2^ obtained through cross-validation was 0.712, while the RF R^2^ obtained on the test dataset was 0.723), again supporting the reliability of the approach selected for model tuning.

Therefore, rather than being discouraging, this limitation represents a huge improvement potential for the future, especially considering the easiness of sequence generation, compared with the difficulties of obtaining a large number of neutralization data or even results of vaccine trials. The availability of a more extensive dataset could provide a noteworthy improvement in our predictive tools as well as a more reliable instrument to test their goodness.

The prompt identification of the best vaccination strategy against NDV is a challenge that field veterinary, companies and health authorities have to face day by day and whose success has a huge economic impact. Currently, vaccination strategies are selected based on personal experience or empirical trials which often lead to sub-optimal and highly diverse schemes. However, field experience suggests that a better antigenic match between vaccine strains and field viruses could significantly improve flock protection [75]. It is also widely recognized that ND vaccines that are phylogenetically closer to circulating field viruses appear to be more effective at reducing NDV shedding and transmission [27,33,76]. Furthermore, the use of antigenically matched inactivated vaccines presents significant safety features, as velogenic vaccine-derived NDVs belonging to genotype III have been described [76].

It is noteworthy that the virus identified in this study as exhibiting the greatest antigenic divergence from vaccine strains (i.e., NDV/chicken/rus/Krasnodar/91/19), was the causative agent of a significant outbreak in Russia that spanned from 2019 to 2021 [75]. It is not possible to exclude that other phenotypic characteristics of this virus or other epidemiological factors were responsible for the magnitude of the epidemic. However, the ability to evade vaccine-induced immunity may have played a role.

The approach here described could provide a rapid and inexpensive tool to wittingly plan vaccination strategies against emerging virus variants based on a scientific and statistical substrate.

Above all, the proposed method expands far beyond veterinary medicine. Potentially any infectious disease can be modeled using this approach if genomic information and a reliable measure of vaccine-induced protection are available from experimental or epidemiological data to train the models. Moreover, the used approaches can be easily adapted to categorical outcome variables (e.g., bivariate outcome: protection/non-protection) which can be more easily obtained in a non-experimental context.

Finally, this computational tool is fully compliant with the policy of the three Rs (Replacement, reduction, and refinement) because it would avoid the use of experimental trials or at least reduce at minimum the number of animals involved, through the pre-selection of a subset of theoretically efficacious vaccines.

## 5. Conclusions

Globally, this study describes a new methodological approach to NDV antigenic distance prediction that, despite the limitation related to the data currently available, provided encouraging results and could be easily improved and extended thanks to more effective data sharing and because of the easiness of sequence and HI data generation. This would be of great benefit to farmers, commercial poultry companies, pharmaceutical industries, and public health authorities potentially leading to relevant improvement in control strategies, economic performances, and animal welfare. Above all, the flexibility of the proposed model allows us to easily extend it to other infectious diseases affecting animals and human beings for which disease prevention and prompt control are even more pressing but for whom ethical issues limit experimental trials.

## Figures and Tables

**Table 1:** Summary of the NDV strain included in this study, genetic characteristics, virulence, and titer of the viral stock produced.

	Strain	Genotype (Dimitrov 2019 [18])	Pathotype ^1^	HA Titer	Titer (EID_50_/100 µL)
Vaccine	VG/GA-AVINEW	I.1.1	Avirulent	1:256	10^8.83^
	V4-like	I.1.2	Avirulent	1:128	10^8.5^
	NDV I2	I.1.1	Avirulent	1:128	10^8.83^
	Ulster	I.2	Avirulent	1:512	10^8.5^
	B1	II	Avirulent	1:512	10^8.5^
	LaSota	II	Avirulent	1:256	10^8.83^
Field virus	APMV-1/Herts_21VIR2596/33	IV	Virulent	1:128	10^8.625^
	APMV-1/chicken/California/18016505-1_19VIR4338/2018	V	Virulent	1:256	10^8.625^
	APMV-1/pigeon/Italy/19VIR8321/2019	VI	Virulent	1:64	10^8^
	APMV-1/chicken/Krasnodar/91_21VIR4521/19	VII.1.1	Virulent	1:128	10^8.5^
	APMV-1/chicken/Romania/19VIR9275-1/2019	VII.1.1	Virulent	1:256	10^9.625^
	APMV-1/broiler/Spain/22VIR7253-24/2022	VII.2	Virulent	1:128	10^8.625^
	APMV-1/chicken/Macedonia/20VIR1984-1/2020	VII.2	Virulent	1:128	10^8.5^
	APMV-1/chicken/Belgium/4096_19RS1-M/2018	VII.2	Virulent	1:64	10^8.625^
	APMV-1/avian/Nigeria/21RS744-46/2021	XIV.2	Virulent	1:64	10^8.625^
	APMV-1/avian/Nigeria/21RS2367-12/2021	XIV.2	Virulent	1:64	10^8.625^
	APMV-1/avian/Nigeria/21RS736-11/2021	XIV.2	Virulent	1:64	10^8.375^
	APMV-1/avian/Nigeria/21RS2368-1/2021	XIV.2	Virulent	1:32	10^8.375^
	APMV-1/avian/Nigeria/21RS2368-6/2021	XIV.2	Virulent	1:32	10^8.375^
	APMV-1/chicken/Cameroon/3490-168_21VIR2562/2008	XVII	Virulent	1:128	10^8.83^
	APMV-1/pigeon/Luxembourg/18175752_18VIR10959/2018	XXI.1.1	Virulent	1:64	10^8.625^
	APMV-1/pigeon/Cyprus/20VIR3543-36_26364-1/2020	XXI.2	Virulent	Not viable	Not viable

^1^: the pathotype was deducted from the F0 cleavage site sequences obtained according to the WOAH terrestrial manual ([39] 2021, Chapter 3.3.14).

**Table 2:** Number of amino acid (AA) differences among the HN (572 amino acid long) and F (554 amino acid long) proteins of the analyzed viruses.

Virus	Genotype	AA Differences to Vaccines HN Gene Level	AA Differences to Vaccines F Gene Level
APMV-1/Herts_21VIR2596/33	IV	34–47	28–48
APMV-1/chicken/California/18016505-1_19VIR4338/2018	V.1	59–70	57–72
APMV-1/pigeon/Italy/19VIR8321/2019	VI.2.1	60–74	49–62
APMV-1/chicken/Krasnodar/91_21VIR4521/19	VII.1.1	56–69	60–74
APMV-1/chicken/Romania/19VIR9275-1/2019	VII.1.1	61-71	48–63
APMV-1/broiler/Spain/22VIR7253-24/2022	VII.2	60–72	44–65
APMV-1/chicken/Macedonia/20VIR1984-1/2020	VII.2	63–75	44–65
APMV-1/chicken/Belgium/4096_19RS1-M/2018	VII.2	60–72	44–64
APMV-1/avian/Nigeria/21RS744-46/2021	XIV.2	68–81	61–78
APMV-1/avian/Nigeria/21RS2367-12/2021	XIV.2	72–84	61–78
APMV-1/avian/Nigeria/21RS736-11/2021	XIV.2	64–75	62–79
APMV-1/avian/Nigeria/21RS2368-1/2021	XIV.2	66–79	59–76
APMV-1/avian/Nigeria/21RS2368-6/2021	XIV.2	66–79	62–79
APMV-1/chicken/Cameroon/3490-168_21VIR2562/2008	XVII	64–74	48–69
APMV-1/pigeon/Luxembourg/18175752_18VIR10959/2018	XXI.1.1	61–73	53–66
APMV-1/pigeon/Cyprus/20VIR3543-36_26364-1/2020	XXI.2	54–70	58–77

Samples received before February 2022.

## Data Availability

All sequences generated and used for data analysis are deposited and openly available on GenBank under access numbers PV137993-PV138014. The raw antigenic data supporting the conclusions of this article will be made available by the authors upon request. The R script has been made publicly available in GitHUB (https://github.com/geofrunz/NDV_ML.git, accessed on 1 March 2025).

## Supplementary Materials

The following supporting information can be downloaded at https://www.mdpi.com/article/10.3390/v17040567/s1, Figure S1: ML phylogenetic tree based on the sequences selected in the present study (highlighted in red) plus the reference dataset provided by [18]. Bootstrap support provided near the corresponding node; Figure S2: Boxplots Performance metrics obtained through cross-validation for different methods and datasets (F, HN, and Merged); Figure S3: Differences in performance parameters between methods pairs and datasets (F, NH, and Merged). The average difference and the confidence interval, corrected for multiple comparisons, indicative of statistical significance, are reported; Table S1: Table reporting the developed ML methods, the optimized hyperparameter, and their respective range of evaluated values. The R packages used are also reported.
