# Supplementary material for: Evaluation of Different Machine Learning Approaches to Predict Antigenic Distance Among Newcastle Disease Virus (NDV) Strains
Source: Viruses. 2025 Apr 14;17(4):567. doi: 10.3390/v17040567 (PMC12031050; doi:10.3390/v17040567)
Supplement: Supplementary file 1 [file viruses-17-00567-s001.zip › Supplementary Figure S1.pdf]

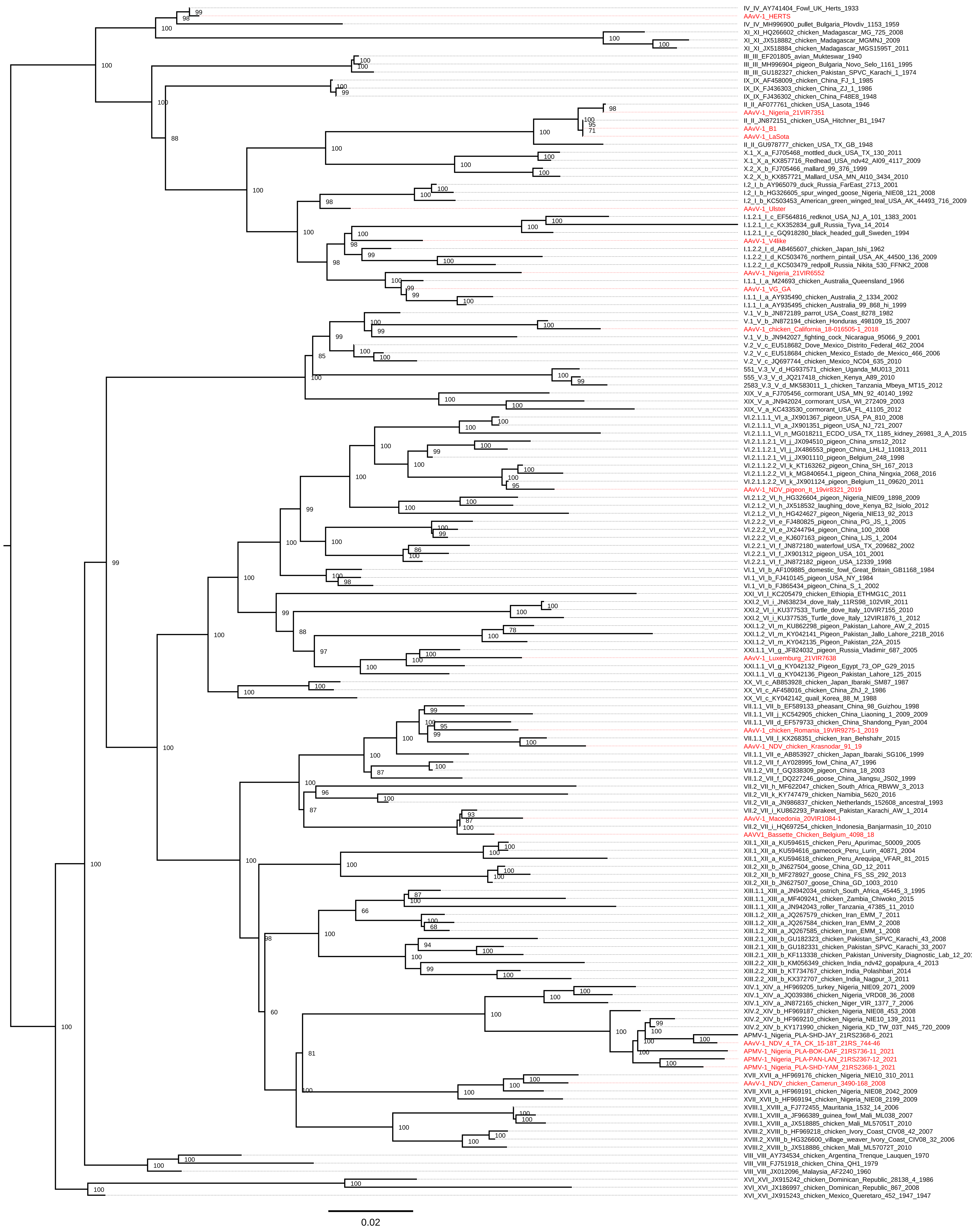
IV_IV_AY741404_Fowl_UK_Herts_1933
AAvV-1_HERTS
IV_IV_MH996900_pullet_Bulgaria_Plovdiv_1153_1959
XI_XI_HQ266602_chicken_Madagascar_MG_725_2008
XI_XI_JX518882_chicken_Madagascar_MGMNJ_2009
XI_XI_JX518884_chicken_Madagascar_MGS1595T_2011
III_III_EF201805_avian_Mukteswar_1940
III_III_MH996904_pigeon_Bulgaria_Novo_Selo_1161_1995
III_III_GU182327_chicken_Pakistan_SPVC_Karachi_1_1974
IX_IX_AF458009_chicken_China_FJ_1_1985
IX_IX_FJ436303_chicken_China_ZJ_1_1986
IX_IX_FJ436302_chicken_China_F48E8_1948
II_II_AF077761_chicken_USA_Lasota_1946
AAvV-1_Nigeria_21VIR7351
II_II_JN872151_chicken_USA_Hitchner_B1_1947
AAvV-1_B1
AAvV-1_LaSota
II_II_GU978777_chicken_USA_TX_GB_1948
X.1_X_a_FJ705468_mottled_duck_USA_TX_130_2011
X.1_X_a_KX857716_Redhead_USA_ndv42_AI09_4117_2009
X.2_X_b_FJ705466_mallard_99_376_1999
X.2_X_b_KX857721_Mallard_USA_MN_AI10_3434_2010
I.2_I_b_AY965079_duck_Russia_FarEast_2713_2001
I.2_I_b_HG326605_spur_winged_goose_Nigeria_NIE08_121_2008
I.2_I_b_KC503453_American_green_winged_teal_USA_AK_44493_716_2009
AAvV-1_Ulster
I.1.2.1_I_c_EF564816_redknot_USA_NJ_A_101_1383_2001
I.1.2.1_I_c_KX352834_gull_Russia_Tyva_14_2014
I.1.2.1_I_c_GQ918280_black_headed_gull_Sweden_1994
AAvV-1_V4like
I.1.2.2_I_d_AB465607_chicken_Japan_Ishi_1962
I.1.2.2_I_d_KC503476_northern_pintail_USA_AK_44500_136_2009
I.1.2.2_I_d_KC503479_redpoll_Russia_Nikita_530_FFNK2_2008
AAvV-1_Nigeria_21VIR6552
I.1.1_I_a_M24693_chicken_Australia_Queensland_1966
AAvV-1_VG_GA
I.1.1_I_a_AY935490_chicken_Australia_2_1334_2002
I.1.1_I_a_AY935495_chicken_Australia_99_868_hi_1999
V.1_V_b_JN872189_parrot_USA_Coast_8278_1982
V.1_V_b_JN872194_chicken_Honduras_498109_15_2007
AAvV-1_chicken_California_18-016505-1_2018
V.1_V_b_JN942027_fighting_cock_Nicaragua_95066_9_2001
V.2_V_c_EU518682_Dove_Mexico_Distrito_Federal_462_2004
V.2_V_c_EU518684_chicken_Mexico_Estado_de_Mexico_466_2006
V.2_V_c_JQ697744_chicken_Mexico_NC04_635_2010
551_V.3_V_d_HG937571_chicken_Uganda_MU013_2011
555_V.3_V_d_JQ217418_chicken_Kenya_A89_2010
2583_V.3_V_d_MK583011_1_chicken_Tanzania_Mbeya_MT15_2012
XIX_V_a_FJ705456_cormorant_USA_MN_92_40140_1992
XIX_V_a_JN942024_cormorant_USA_WI_272409_2003
XIX_V_a_KC433530_cormorant_USA_FL_41105_2012
VI.2.1.1.1_VI_a_JX901367_pigeon_USA_PA_810_2008
VI.2.1.1.1_VI_a_JX901351_pigeon_USA_NJ_721_2007
VI.2.1.1.1_VI_n_MG018211_ECDO_USA_TX_1185_kidney_26981_3_A_2015
VI.2.1.1.2.1_VI_j_JX094510_pigeon_China_sms12_2012
VI.2.1.1.2.1_VI_j_JX486553_pigeon_China_LHLJ_110813_2011
VI.2.1.1.2.1_VI_j_JX901110_pigeon_Belgium_248_1998
VI.2.1.1.2.2_VI_k_KT163262_pigeon_China_SH_167_2013
VI.2.1.1.2.2_VI_k_MG840654.1_pigeon_China_Ningxia_2068_2016
VI.2.1.1.2.2_VI_k_JX901124_pigeon_Belgium_11_09620_2011
AAvV-1_NDV_pigeon_It_19vir8321_2019
VI.2.1.2_VI_h_HG326604_pigeon_Nigeria_NIE09_1898_2009
VI.2.1.2_VI_h_JX518532_laughing_dove_Kenya_B2_Isiolo_2012
VI.2.1.2_VI_h_HG424627_pigeon_Nigeria_NIE13_92_2013
VI.2.2.2_VI_e_FJ480825_pigeon_China_PG_JS_1_2005
VI.2.2.2_VI_e_JX244794_pigeon_China_100_2008
VI.2.2.2_VI_e_KJ607163_pigeon_China_LJS_1_2004
VI.2.2.1_VI_f_JN872180_waterfowl_USA_TX_209682_2002
VI.2.2.1_VI_f_JX901312_pigeon_USA_101_2001
VI.2.2.1_VI_f_JN872182_pigeon_USA_12339_1998
VI.1_VI_b_AF109885_domestic_fowl_Great_Britain_GB1168_1984
VI.1_VI_b_FJ410145_pigeon_USA_NY_1984
VI.1_VI_b_FJ865434_pigeon_China_S_1_2002
XXI_VI_l_KC205479_chicken_Ethiopia_ETHMG1C_2011
XXI.2_VI_i_JN638234_dove_Italy_11RS98_102VIR_2011
XXI.2_VI_i_KU377533_Turtle_dove_Italy_10VIR7155_2010
XXI.2_VI_i_KU377535_Turtle_dove_Italy_12VIR1876_1_2012
XXI.1.2_VI_m_KU862298_pigeon_Pakistan_Lahore_AW_2_2015
XXI.1.2_VI_m_KY042141_Pigeon_Pakistan_Jallo_Lahore_221B_2016
XXI.1.2_VI_m_KY042135_Pigeon_Pakistan_22A_2015
XXI.1.1_VI_g_JF824032_pigeon_Russia_Vladimir_687_2005
AAvV-1_Luxemburg_21VIR7638
XXI.1.1_VI_g_KY042132_Pigeon_Egypt_73_OP_G29_2015
XXI.1.1_VI_g_KY042136_Pigeon_Pakistan_Lahore_125_2015
XX_VI_c_AB853928_chicken_Japan_Ibaraki_SM87_1987
XX_VI_c_AF458016_chicken_China_ZhJ_2_1986
XX_VI_c_KY042142_quail_Korea_88_M_1988
VII.1.1_VII_b_EF589133_pheasant_China_98_Guizhou_1998
VII.1.1_VII_j_KC542905_chicken_China_Liaoning_1_2009_2009
VII.1.1_VII_d_EF579733_chicken_China_Shandong_Pyan_2004
AAvV-1_chicken_Romania_19VIR9275-1_2019
VII.1.1_VII_l_KX268351_chicken_Iran_Behshahr_2015
AAvV-1_NDV_chicken_Krasnodar_91_19
VII.1.1_VII_e_AB853927_chicken_Japan_Ibaraki_SG106_1999
VII.1.2_VII_f_AY028995_fowl_China_A7_1996
VII.1.2_VII_f_GQ338309_pigeon_China_18_2003
VII.1.2_VII_f_DQ227246_goose_China_Jiangsu_JS02_1999
VII.2_VII_h_MF622047_chicken_South_Africa_RBWW_3_2013
VII.2_VII_k_KY747479_chicken_Namibia_5620_2016
VII.2_VII_a_JN986837_chicken_Netherlands_152608_ancestral_1993
VII.2_VII_i_KU862293_Parakeet_Pakistan_Karachi_AW_1_2014
AAvV-1_Macedonia_20VIR1084-1
VII.2_VII_i_HQ697254_chicken_Indonesia_Banjarmasin_10_2010
AAVV1_Bassette_Chicken_Belgium_4098_18
XII.1_XII_a_KU594615_chicken_Peru_Apurimac_50009_2005
XII.1_XII_a_KU594616_gamecock_Peru_Lurin_40871_2004
XII.1_XII_a_KU594618_chicken_Peru_Arequipa_VFAR_81_2015
XII.2_XII_b_JN627504_goose_China_GD_12_2011
XII.2_XII_b_MF278927_goose_China_FS_SS_292_2013
XII.2_XII_b_JN627507_goose_China_GD_1003_2010
XIII.1.1_XIII_a_JN942034_ostrich_South_Africa_45445_3_1995
XIII.1.1_XIII_a_MF409241_chicken_Zambia_Chiwoko_2015
XIII.1.1_XIII_a_JN942043_roller_Tanzania_47385_11_2010
XIII.1.2_XIII_a_JQ267579_chicken_Iran_EMM_7_2011
XIII.1.2_XIII_a_JQ267584_chicken_Iran_EMM_2_2008
XIII.1.2_XIII_a_JQ267585_chicken_Iran_EMM_1_2008
XIII.2.1_XIII_b_GU182323_chicken_Pakistan_SPVC_Karachi_43_2008
XIII.2.1_XIII_b_GU182331_chicken_Pakistan_SPVC_Karachi_33_2007
XIII.2.1_XIII_b_KF113338_chicken_Pakistan_University_Diagnostic_Lab_12_20
XIII.2.2_XIII_b_KM056349_chicken_India_ndv42_gopalpura_4_2013
XIII.2.2_XIII_b_KT734767_chicken_India_Polashbari_2014
XIII.2.2_XIII_b_KX372707_chicken_India_Nagpur_3_2011
XIV.1_XIV_a_HF969205_turkey_Nigeria_NIE09_2071_2009
XIV.1_XIV_a_JQ039386_chicken_Nigeria_VRD08_36_2008
XIV.1_XIV_a_JN872165_chicken_Niger_VIR_1377_7_2006
XIV.2_XIV_b_HF969187_chicken_Nigeria_NIE08_453_2008
XIV.2_XIV_b_HF969210_chicken_Nigeria_NIE10_139_2011
XIV.2_XIV_b_KY171990_chicken_Nigeria_KD_TW_03T_N45_720_2009
APMV-1_Nigeria_PLA-SHD-JAY_21RS2368-6_2021
AAvV-1_NDV_4_TA_CK_15-18T_21RS_744-46
APMV-1_Nigeria_PLA-BOK-DAF_21RS736-11_2021
APMV-1_Nigeria_PLA-PAN-LAN_21RS2367-12_2021
APMV-1_Nigeria_PLA-SHD-YAM_21RS2368-1_2021
XVII_XVII_a_HF969176_chicken_Nigeria_NIE10_310_2011
AAvV-1_NDV_chicken_Camerun_3490-168_2008
XVII_XVII_b_HF969191_chicken_Nigeria_NIE08_2042_2009
XVII_XVII_b_HF969194_chicken_Nigeria_NIE08_2199_2009
XVIII.1_XVIII_a_FJ772455_Mauritania_1532_14_2006
XVIII.1_XVIII_a_JF966389_guinea_fowl_Mali_ML038_2007
XVIII.1_XVIII_a_JX518885_chicken_Mali_ML57051T_2010
XVIII.2_XVIII_b_HF969218_chicken_Ivory_Coast_CIV08_42_2007
XVIII.2_XVIII_b_HG326600_village_weaver_Ivory_Coast_CIV08_32_2006
XVIII.2_XVIII_b_JX518886_chicken_Mali_ML57072T_2010
VIII_VIII_AY734534_chicken_Argentina_Trenque_Lauquen_1970
VIII_VIII_FJ751918_chicken_China_QH1_1979
VIII_VIII_JX012096_Malaysia_AF2240_1960
XVI_XVI_JX915242_chicken_Dominican_Republic_28138_4_1986
XVI_XVI_JX186997_chicken_Dominican_Republic_867_2008
XVI_XVI_JX915243_chicken_Mexico_Queretaro_452_1947_1947
0.02
