# Supplementary material for: Evaluation of Different Machine Learning Approaches to Predict Antigenic Distance Among Newcastle Disease Virus (NDV) Strains
Source: Viruses. 2025 Apr 14;17(4):567. doi: 10.3390/v17040567 (PMC12031050; doi:10.3390/v17040567)
Supplement: Supplementary file 1 [file viruses-17-00567-s001.zip › Supplementary Table S1.pdf]

Method	***Parametes***	***Range***	***Package***
Random Forest	mtry (Number of Randomly Selected Predictors)	Mtry = 1 to (number of features/3)	*randomForest*
Tree bagging	B (Bootstrap number)	500	*treebag*
Artificial Neural Network	Layer (Number of hidden Units in Layer 1 and 2)	layer1 =1 to10; layer2=0 to 5	*neuralnet*
Support Vector Machine	Kind of kernel	Linear/Radial	*svmLinear*
	C (Cost)<br>S (Sigma)	C =0.01 to 70; S = 0.01 to 10;	*svmRadial*

Supplementary Table S1. Table reporting the developed ML methods, the optimized hyperparameter and their respective range of evaluated values. The R packages used is also reported.
