# Supplementary figures and images for: Evaluation of Different Machine Learning Approaches to Predict Antigenic Distance Among Newcastle Disease Virus (NDV) Strains
Source: Viruses. 2025 Apr 14;17(4):567. doi: 10.3390/v17040567 (PMC12031050; doi:10.3390/v17040567)

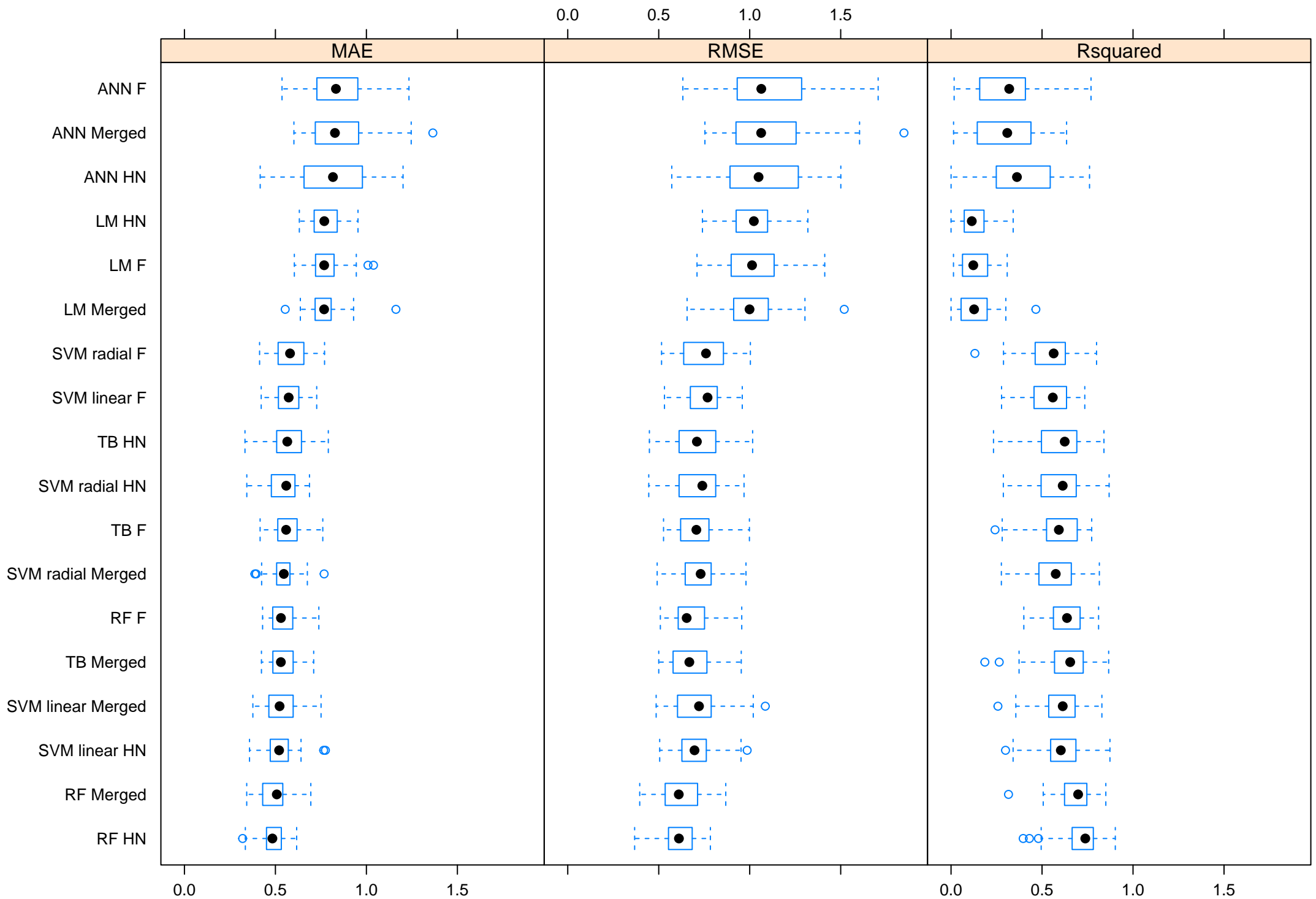

Supplement: Supplementary file 1 [file viruses-17-00567-s001.zip › Supplementary Figure S2.pdf]
