# Supplementary material for: Evaluation of Different Machine Learning Approaches to Predict Antigenic Distance Among Newcastle Disease Virus (NDV) Strains
Source: Viruses. 2025 Apr 14;17(4):567. doi: 10.3390/v17040567 (PMC12031050; doi:10.3390/v17040567)
Supplement: Supplementary file 1 [file Supplementary Figure S3.pdf › Supplementary Figure S3.pdf]

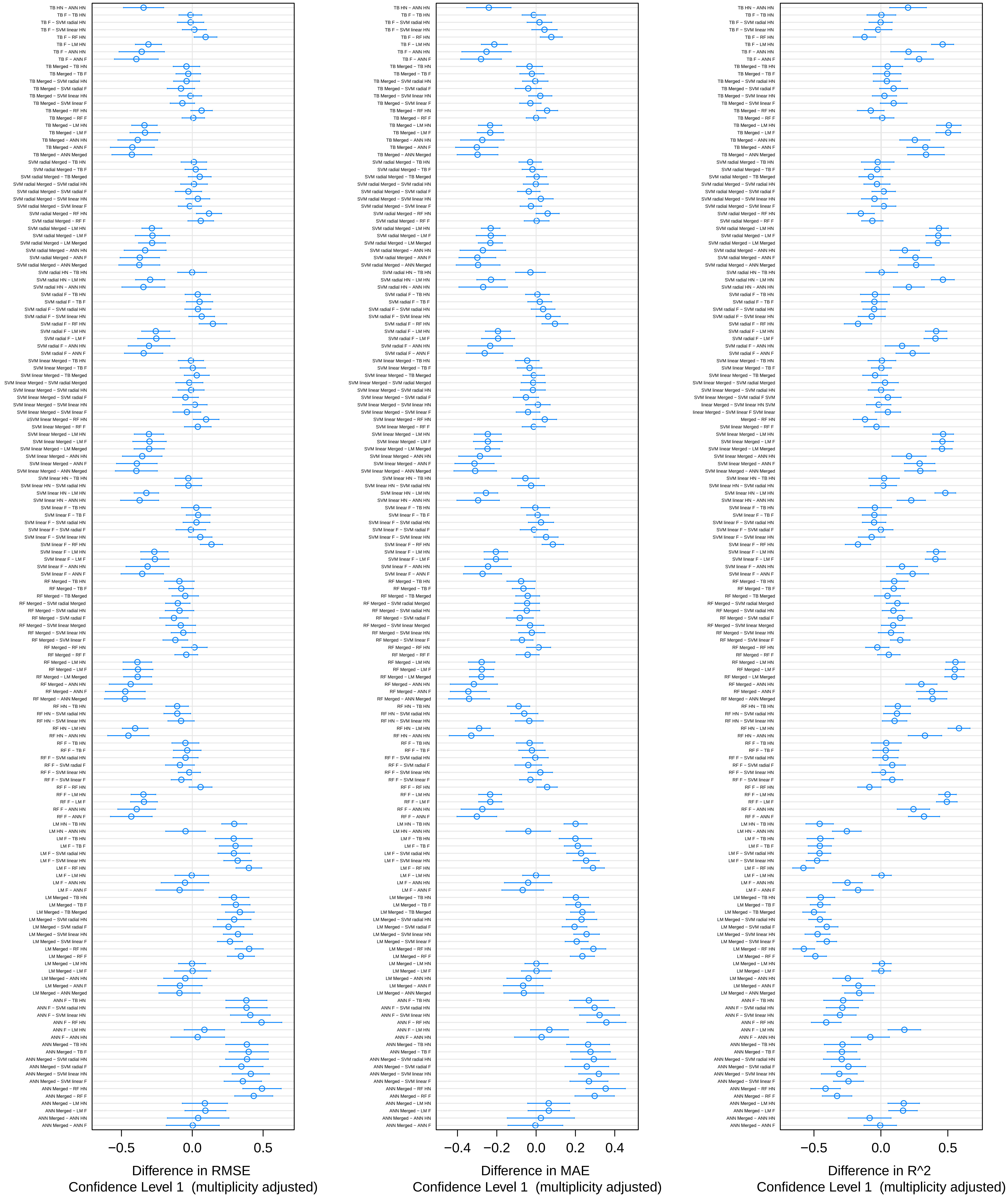
−0.5
0.0
0.5
Difference in RMSE
Confidence Level 1 (multiplicity adjusted)
−0.4
−0.2
0.0
0.2
0.4
Difference in MAE
Confidence Level 1 (multiplicity adjusted)
−0.5
0.0
0.5
Difference in R^2
Confidence Level 1 (multiplicity adjusted)
